# Diagnosis of thyroid nodules for ultrasonographic characteristics indicative of malignancy using random forest

**DOI:** 10.1186/s13040-020-00223-w

**Published:** 2020-09-03

**Authors:** Dan Chen, Jun Hu, Mei Zhu, Niansheng Tang, Yang Yang, Yuran Feng

**Affiliations:** 1 Yunnan Key Laboratory of Statistical Modeling and Data Analysis, Yunnan University, Kunming, 650091 China; 2 Department of Ultrasound, The First Affiliated Hospital of Kunming Medical University, Kunming, 650032 China; 3 College of Science, Yunnan Agricultural University, Kunming, 650201 China

**Keywords:** Random forest, Risk score, Thyroid nodule, Ultrasonographic characteristic

## Abstract

**Background:**

Various combinations of ultrasonographic (US) characteristics are increasingly utilized to classify thyroid nodules. But they lack theories, and heavily depend on radiologists’ experience, and cannot correctly classify thyroid nodules. Hence, our main purpose of this manuscript is to select the US characteristics significantly associated with malignancy and to develop an efficient scoring system for facilitating ultrasonic clinicians to correctly identify thyroid malignancy.

**Methods:**

A logistic regression (LR) model is utilized to identify the potential thyroid malignancy, and the least absolute shrinkage and selection operator (LASSO) method is adopted to simultaneously select US characteristics significantly associated with malignancy and estimate parameters in LR model. Based on the selected US characteristics, we calculate the probability for each of thyroid nodules via random forest (RF) and extreme learning machine (ELM), and develop a scoring system to classify thyroid nodules. For comparison, we also consider eight state-of-the-art methods such as support vector machine (SVM), neural network (NET), etc. The area under the receiver operating characteristic curve (AUC) is employed to measure the accuracy of various classifiers.

**Results:**

The US characteristics: nodule size, AP/T≥1, solid component, micro-calcifications, hackly border, hypoechogenicity, presence of halo, unclear border, irregular margin, and central vascularity are selected as the significant predictors associated with thyroid malignancy via the LASSO LR (LLR). Using the developed scoring system, thyroid nodules are classified into the following four categories: benign, low suspicion, intermediate suspicion, and high suspicion, whose rates of malignancy correctly identified for RF (ELM) method on the testing dataset are 0.0% (4.3%), 14.3% (50.0%), 58.1% (59.1%) and 96.1% (97.7%), respectively.

**Conclusion:**

LLR together with RF performs better than other methods in identifying malignancy, especially for abnormal nodules, in terms of risk scores. The developed scoring system can well predict the risk of malignancy and guide medical doctors to make management decisions for reducing the number of unnecessary biopsies for benign nodules.

## Background

With the development of new ultrasound technology and the popularity of high-resolution scanners, it is no longer challenging to detect thyroid nodules. However, for most sonographers, the critical challenge is to distinguish both malignant thyroid nodules and benign ones. To this end, some US characteristics, such as the presence of unclear border, micro-calcifications, irregular shape, solid component, inner echo [1–3], are widely adopted to assess nodules at risk for malignancy. Some studies have shown that only using one of the US characteristics mentioned above is impossible to correctly distinguish between malignant nodules and benign ones [4]. Many malignant nodules usually have more than two representative characteristics. Therefore, it may be rather desirable to develop an efficient approach to improve the diagnostic accuracy for thyroid malignancy by incorporating multiple characteristics mentioned above. On the other hand, the US examination can provide many potential characteristics, but some of them are inactive for the diagnosis of thyroid cancer. Thus, distinguishing inactive characteristics and active ones may largely improve the accuracy of the diagnosis of thyroid malignancy.

In previous studies [1, 5–11], different versions of thyroid imaging reporting and data systems (TI-RADS) were proposed for thyroid nodule diagnosis and management by considering different combinations of US characteristics. Although these systems can be used to improve the efficiency of thyroid nodule diagnosis compared with the traditional subjective diagnosis, they did not provide a quantitative approach to assess the risk of the malignant tumor. To this end, in 2017, the American college of radiology (ACR) published an ACR system [11] for estimating the risk of malignancy, in which TI-RADS scores were calculated from 5 categories of US characteristics. For this, the ACR TI-RADS has been widely applied to thyroid nodule diagnosis now. However, the cumulative score calculated from 5 categories of US characteristics still heavily relies on the radiologist’s description for the used characteristics, and the efficiency of the scoring system varies with the radiologist’s experience. Moreover, existing approaches mentioned above sometimes behave poorly and lack theories. To overcome these defects, in a recent study [12], machine learning algorithms such as random forest (RF), kernel support vector machine (SVM), neural network (NET), etc., were introduced to classify thyroid nodules into two kinds: benign and malign based on the used US characteristics. But they did not consider which US characteristics were active and which ones were inactive in detecting malign. Moreover, they only considered two types of thyroid nodules, which were impossible for patients to understand the phase of thyroid nodules. To our knowledge, there is little work on the scoring system developed by RF in the differentiated diagnosis of thyroid nodules. Hence, the main purpose of this paper is to develop an objective and quantitative scoring system to assist ultrasonic clinicians for identifying the thyroid cancer by (i) adopting a LASSO method to efficiently select the critical US characteristics significantly associated with malignancy as potential predictors of malignancy; (ii) using machine learning algorithms to calculate the class probability of each nodule, which is utilized to classify for each nodule; (iii) proposing a scoring system that can be used to predict the risk for malignancy and guide medical doctors to make management decisions for reducing the number of unnecessary biopsies for benign nodules.

## Materials and methods

### Thyroid nodules

Consider a dataset with 1558 thyroid nodules for 1480 patients collected during the period from Jan. 2011 to Apr. 2016 at The First Affiliated Hospital of Kunming Medical University in China. In this dataset, 110 thyroid nodules for 110 patients (94 females and 16 males) can be regarded as outliers detected by traditional LR analysis and test for score [13]. Among these outliers, 68.7% (68/99) of benign nodules has at least 3 known US malignancy characteristics, and 72.7% (8/11) malignant nodules has at least four benign characteristics. It is difficult to differentiate between malignant and benign nodules for these outliers only based on the US characteristics selected by existing methods [13]. Therefore, these outliers are regarded as abnormal nodules, and the remaining 1448 nodules for 1370 patients (286 male and 1084 female) are deemed as disease nodules. Among 1370 patients, the oldest patient is 80 years old, and the youngest patient is only 10 years old. Surgery has been performed on all the nodules. Table 1 presents the numbers of begin and malignant nodules for female and male groups, respectively, and means of patients’ ages for disease and abnormal nodules, respectively. Examination of Table 1 shows that (i) malignant patients are younger than benign patients in disease nodule group, (ii) benign patients are younger than malignant ones in abnormal nodule group. Although age is not a US characteristic in traditional diagnosis of thyroid nodules, these observations indicate that age may be an important factor associated with the malignant.

**Table 1:** Age and gender distribution of cases in disease nodules and abnormal nodules

	Cytology	No. of nodules	Female	Male	Mean Age ±SD
Disease nodules	Benign	248	189	43	57.2±10.7
	Malignant	1200	895	243	43.1±11.4
Abnormal nodules	Benign	99	85	14	46.2±10.9
	Malignant	11	9	2	56.5±9.9

Abbreviations: SD represents standard deviation

### US characteristics

**Fig. 1:**
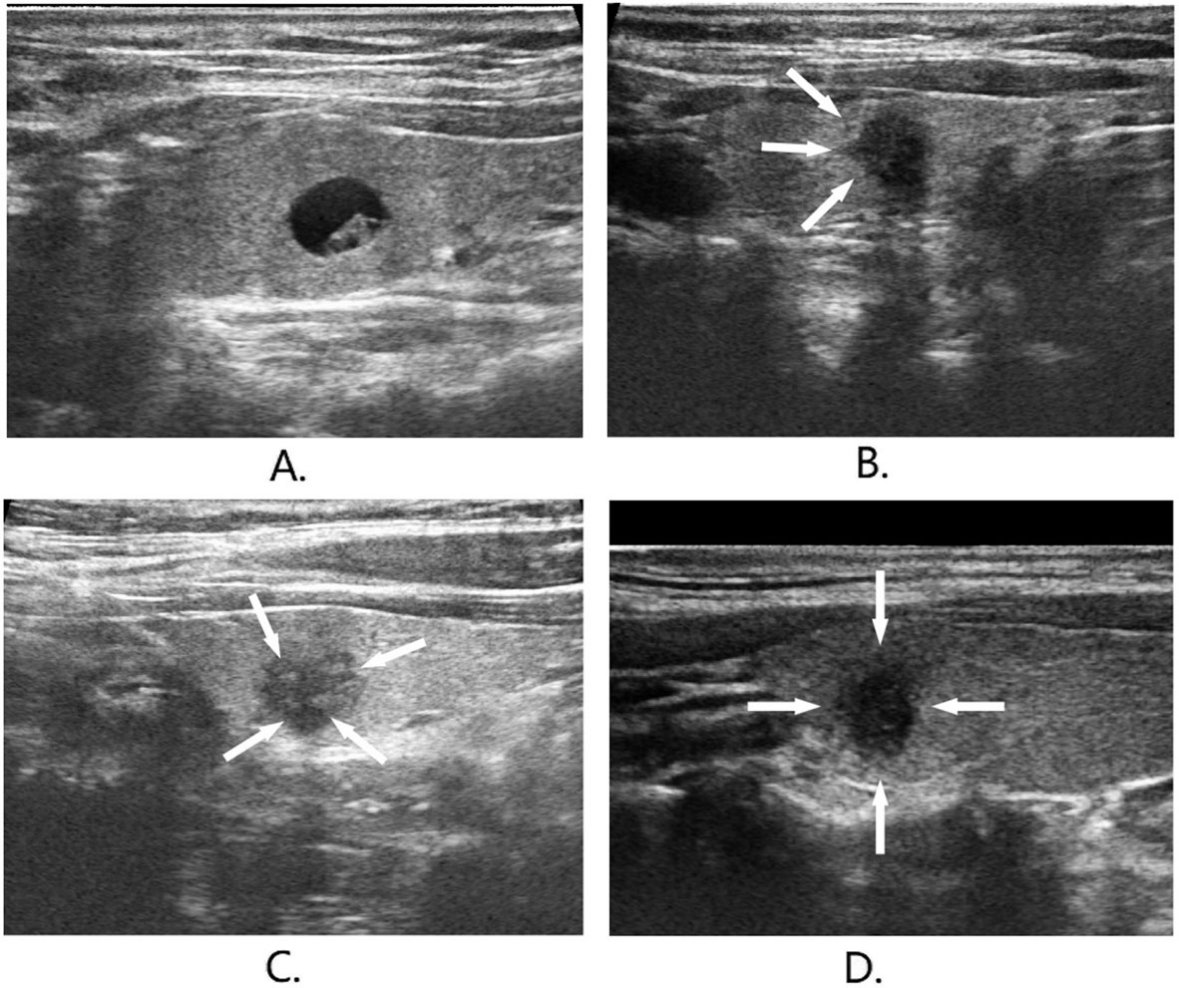
US scans show characteristics of thyroid modules: **a** beign nodules; **b** malignant nodule with hackly border; **c** malignant nodule with micro-calcifications; **d** malignant nodule with (AP/T ≥1) and irregular margin

We used GE LOGIQ E9 and HITACHI for ultrasonic scanning and performed thyroid area scanning with a linear array probe. The type of probe was ML6-15. To ensure the comparability of thyroid images in all patients, we kept the frequency of the parameter at 10MHz. The real-time US was performed by five physicians. Incorporating various studies, we consider the following US characteristics: margin (regular and irregular), border (unclear or clear), hackly border (present or absent), halo (present or absent), vascularity (peripheral, mixed, central), blood flow degree (low, medium, high), posterior echo attenuation (present or absent), lateral shadow (present or absent), echogenicity (hypoechoic or hyperechoic), calcification (micro-calcifications, macro-calcifications or none-calcifications), shape (AP/T ≥1 or AP/T <1) defined as the shape ratio (i.e., the ratio of the anteroposterior diameter of the nodule to the transverse diameter). Component (solid or mixed) was defined in terms of the ratio of the cystic portion to the solid portion as solid and mixed (e.g., see Fig. 1). The size and age of nodules are here considered.

### Analysis of hypoechogenicity

Several studies [14–16] have pointed out that hypoechogenicity is a highly suspicious characteristic of malignancy. Moreover, echogenicity can be measured by the echogenicity ratio (ER), which is defined as the ratio of the echogenicity of the nodule to the anterior cervical muscles. Echogenicity is usually classified into the following two categories: hypoechogenicity and hyperechogenicity. If the ER is less than or equal to some cutoff, it is taken as hypoechogenicity; otherwise, it is defined as hyperechogenicity.

To determine the best cutoff, we calculate the area under the receiver operating characteristic curve (AUC) at all the observed cutoffs ranging from 0.0 to 5.0. The cutoff corresponding to the maximum of AUC values is regarded as the optimal cutoff. The AUC is widely utilized as a measure of the performance of classifiers in machine learning, and is a better measure than Matthews correlation coefficient for assessing the prediction accuracy of a classifier in the imbalanced dataset.

### Selection of US characteristics

Let *y*_*i*_ be a binary response variable, i.e., *y*_*i*_=0 if the *i*-th nodule is benign, and *y*_*i*_=1 if the *i*-th nodule is malignant, and *X*_*i*_=(*x*_*i*1_,*x*_*i*2_,…,*x*_*im*_)^⊤^ be a vector of US characteristics associated with the *i*-th nodule. The ordinary logistic regression (LR) for response *y*_*i*_ has the form

1 $$ \text{Pr}\left(y_{i}=1|X_{i}\right)=\frac{\exp\left(\beta_{0}+\sum_{j=1}^{m}\beta_{j} x_{ij}\right)}{1+\exp\left(\beta_{0}+\sum_{j=1}^{m}\beta_{j} x_{ij}\right)},  $$

where *β*_0_ is an intercept, and *β*_1_,…,*β*_*m*_ are regression coefficients. The nodule with the probability tending to 1 is regarded as a malignant nodule, while the nodule with the probability tending to 0 is taken as a benign nodule. It is widely recognized that the above considered LR model may be subject to the overfitting problem due to some inactive covariates encompassed. To address this issue, the best subset selection method, such as the Akaike information criterion (AIC) and Bayesian information criterion (BIC), can be used to select active covariates. However, it was a multi-step method. Thus, it is quite time-consuming when the number of covariates is moderate (e.g., [16]) or large. To solve the aforementioned problem, a well-known LASSO method [17] is employed to simultaneously estimate regression coefficients and select active US characteristics in the above considered LR model in that it is a regularization procedure that shrinks regression coefficients toward zero, and can simplify the model via variable selection procedure.

Estimators of parameters *β*_0_,*β*_1_,…,*β*_*m*_ can be obtained by maximizing the following penalized log-likelihood function:

2 $$ \sum_{i=1}^{n}\left\lbrace \sum_{j=1}^{m}y_{i}\left(\beta_{0}+\beta_{j} x_{ij} \right) -\log\left[1+\exp\left(\beta_{0}+\sum_{j=1}^{m}\beta_{j} x_{ij}\right) \right] \right\rbrace -\lambda\sum_{j=1}^{m}\left|\beta_{j}\right|  $$

where *λ*≥0 is a tuning parameter to be estimated. When *λ* is sufficiently large, some of parameter estimates are forced to be exactly zero [18–20].

**Fig. 2:**
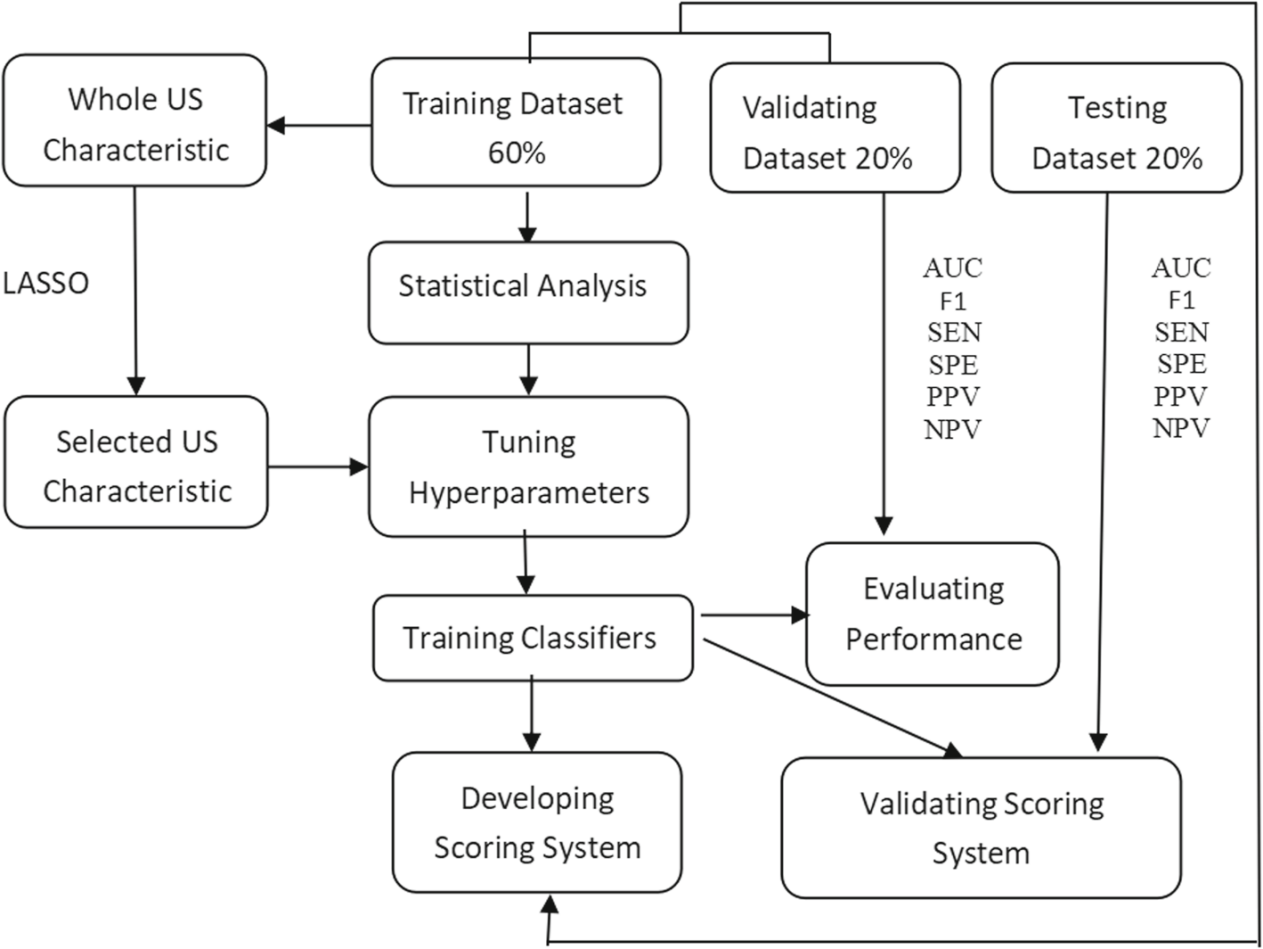
Flow chart of our proposed method

As is shown in Fig. 2, we randomly divided the whole dataset into 60% for the training dataset, 20% for the validating dataset, and 20% for the testing dataset using the stratified sampling technique. The LASSO shrinkage parameter *λ* (lambda.1se) is selected by the mean of 10-fold cross-validation using the glmnet R package for the training dataset. We then estimate parameters *β*_*j*_’s and select active US characteristics as those whose corresponding estimated parameters are not equal to zero, and take the model with maximizing AUC as the best model, where the result of the surgery is regarded as the gold standard of reference. The US characteristics with nonzero estimated parameters in the best model are retained as active predictors.

Note that the US characteristics selected above are random since the considered dataset is randomly divided into training and testing data. To address the issue, we repeat the above-presented procedure 100 times and then retain the US characteristics that occur with the largest frequencies among 100 repetitions.

### Scoring for nodules

Data were analyzed using R version 3.6.1 (2019-07-05), R packages [20] were used for each of the following classified methods: randomForest(·) (randomForest, RF: Random Forest), glm(·) (glmnet, LR: Logistic Regression), ksvm(·) (e1071, SVM: Support Vector Machine), nnet(·) (nnet, NET: neural network), elm_train(·) (elmNNRcpp, ELM: Extreme learning machine), kknn(·) (kknn, KNN: k-nearest neighborhood), naiveBayes(·) (e1071, NB: Naive Bayesian), boosting(·) (adabag, ADAB: Adaptive boosting), LiblineaR(·) (LiblineaR, LOG: L^2^-logistic regression), lda(·) (MASS, LDA: Linear discriminant analysis), respectively.

RF is an ensemble classifier that consists of many decision trees. Each decision tree is a classifier for classification. To classify an input sample, *N* trees have *N* classification results. The RF integrates all voting results and takes the class with the most voting times as the final output. At each tree split, a random sample of features is selected, and the tree is only allowed to split on those selected feature directions. Here, the “randomForest” function is the classification, and regression tree (CART) uses the Gini impurity criterion as a feature selection measure to construct a decision tree.

We train the classifiers on the training dataset mentioned above. When the training data are fed through the RF, a class probability (i.e., the level of risk) *P*_RF_ that is the percentage of trees voted for the malignant nodules is outputted. Thus, a thyroid nodule is identified as malignant with probability *P*_RF_ and benign with probability 1−*P*_RF_. For comparison, we also compute the results using a LR model, extreme learning machine (ELM) [21, 22] as well as the state-of-the-art methods (e.g., SVN, NET, KNN, NB, ADAB, LOG and LDA) discussed by Zhang et al. [12]. Again, to eliminate randomness, we repeat the above-presented partition 100 times, leading to 100 classifier sets. The risk score *S*_RF_ of malignancy for each of thyroid nodules is defined as the averaged class probability for 100 repetitions. The risk scores corresponding to LR, SVM, NET, ELM, KNN, NB, ADAB, LOG and LDA methods are denoted as *S*_LR_, *S*_SVM_, *S*_NET_, *S*_ELM_, *S*_KNN_, *S*_NB_, *S*_ADAB_, *S*_LOG_ and *S*_LDA_, respectively.

Here, the Caret package (e.g., https://cran.r-project.org/web/packages/caret/vignettes/\\caret.html) is employed to tune hyperparameters for all classifiers via 10-fold cross-validation on the training dataset based on the selection of the following parameters: ntree=500 and mtry=2, where grid search is conducted to tune hyper-parameter mtry. At the same time, a grid search method is utilized to optimize the corresponding parameters of SVM (sigma=0.071, and C=0.25, kernel=Radial Basis kernel), NET (size=1, decay=0.1), EL*M*_train_(nhid=50,actfun=sig), KNN(kernel=“rectangular”, k=9), NB(laplace=2), Boosting(boos = TRUE, mfinal=100, coeflearn= Breiman), LiblineaR(type=0, bias= “TRUE”, verbose= “FALSE"”) and LDA (method= mle).

## Results

### Hypoechogenicity

**Fig. 3:**
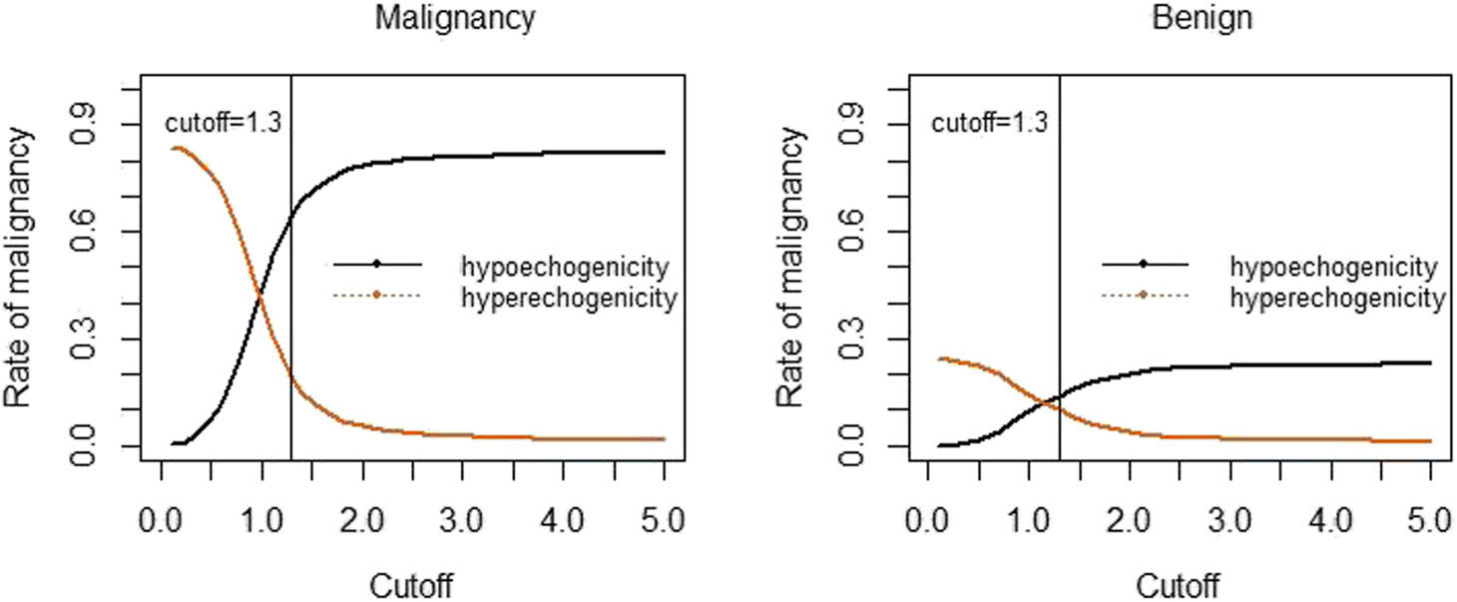
Cutoff of echogenicity ratio vs. rate of malignancy. The vertical line corresponds to the cutoff =1.3

**Fig. 4:**
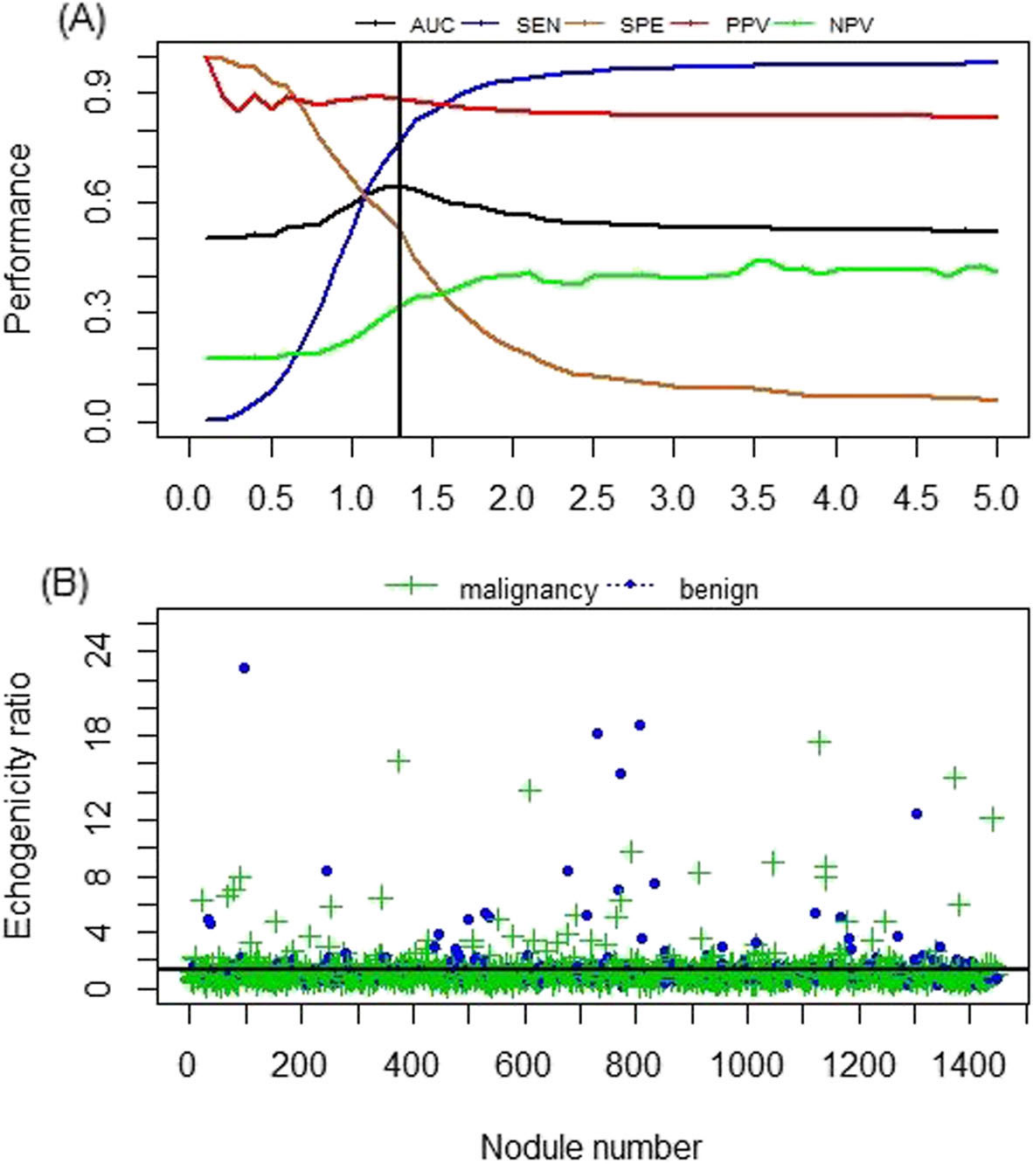
Cutoff and performance of hypoechogenicity in the diagnosis of malignant nodules. Scatter diagram of ER distribution for benign and malignant thyroid nodules. The vertical line in **a** and horizontal line in **b** correspond to the cutoff = 1.3, respectively

Hypoechogenicity does not mean that the echogenicity ratio (ER) is as low as possible. The previous studies show that hypoechogenicity is associated with the increased malignancy risk [3, 14]. It is easily seen from Fig. 3 that the rate of malignancy for hyperechoic nodules is much higher than that of hypoechoic nodules regardless of malignancy and benign nodules when the cutoff is less than 0.9. In contrast, when the cutoff is larger than 2.4, the rate of malignancy for hypoechoic nodules increases slowly regardless of malignancy and benign nodules. The optimal cutoff of ER should be taken so that hypoechogenicity has a good diagnostic performance of differentiating malignant and benign nodules. Figure 4 depicts the performance at each cutoff of the ER. Examination of Fig. 4 implies that the optimal cutoff should be taken as 1.3 because the AUC attains the maximum 0.65 at cutoff=1.3; and for the optimal cutoff, hypoechogenicity has the sensitivity (SEN) 76.7%, the specificity (SPE) 52.8%, the positive predictive value (PPV) 88.7%, and the negative predictive value (NPV) 31.9% when detecting malignant nodules. On the other hand, for the malignant (or benign) cases, 76.7% (or 47.2%) is hypoechoic (e.g., see Fig. 3), leading to the conclusion that there is a significant difference between the malignant and benign nodules for hypoechogenicity due to the *p*-value (*P*<0.001) calculated from Fisher’s exact test method. The above fact shows that using the optimal cutoff=1.3 to distinguish both malignant and benign nodules can yield good diagnostic performance.

### US characteristic selection

**Fig. 5:**
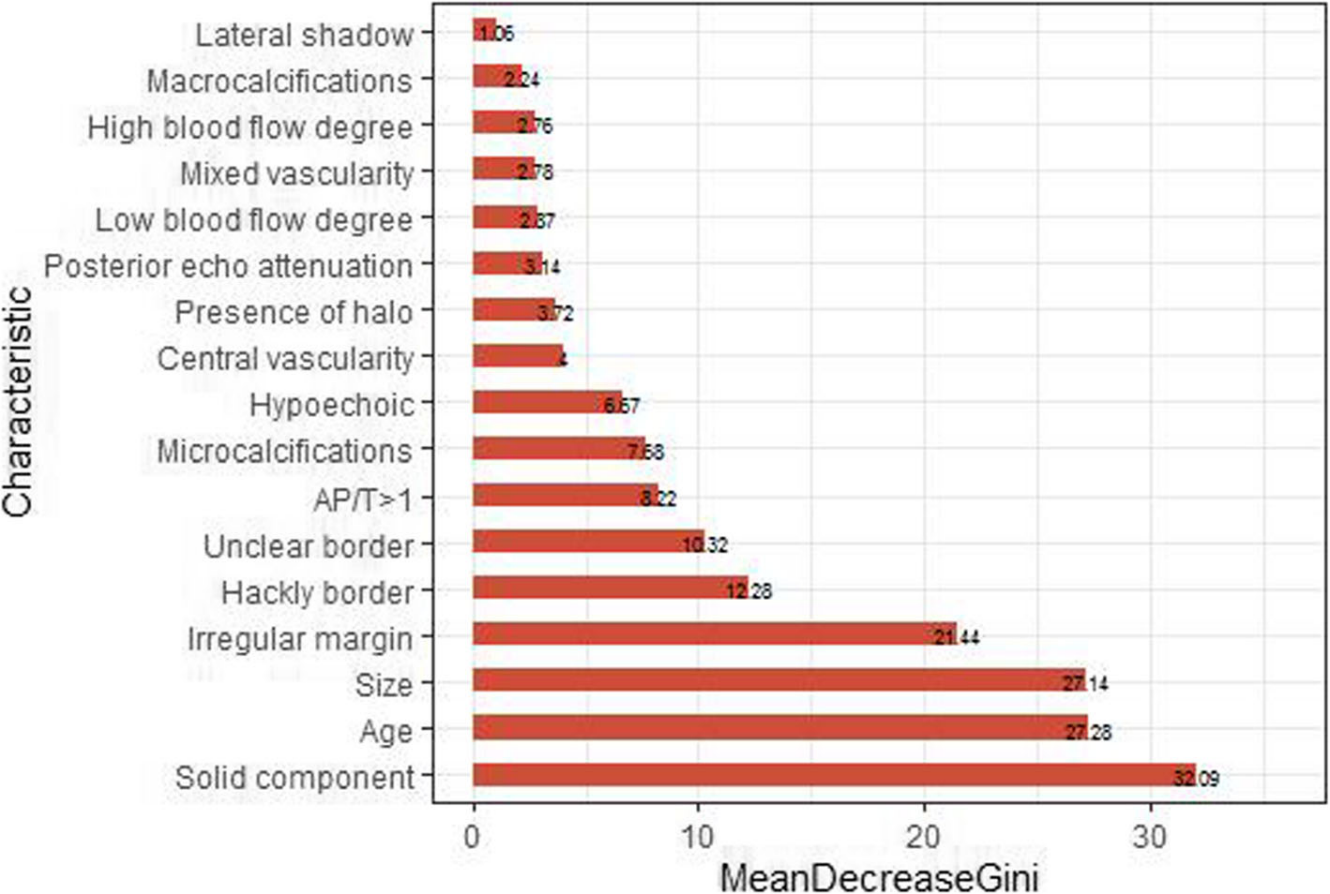
Importance of US characteristics by RF

From LLR analysis, characteristics: nodule size, AP/T≥1, solid component, micro- calcifications, hackly border, hypoechogenicity, presence of halo, unclear border, irregular margin and central vascularity are selected as active predictors associated with malignancy. Table 2 reports the diagnostic performance for each of the US characteristics in terms of the prediction of malignancy. Examination of Table 2 shows that the selected characteristics have relatively high NPV (23.5%–76.5%), SEN (57%–96.4%), and AUC (0.547–0.776) compared to those not selected with NPV (15.6%–20.3%), SEN (4.3% – 34.8%) and AUC (0.456–0.575). Among the selected characteristics, irregular margin (NPV: 49.2%, SEN: 85.0%) achieves the highest AUC (0.776). Central vascularity identified by spectral Doppler US is also selected as a malignant characteristic, even though some studies suggest that the increased central vascularity is not reliable for the malignant evaluation of thyroid nodule, and other authors pointed out that the increased central vascularity is accepted as a supporting characteristic for diagnosis of malignancy [23]. Nodule size and age are detected as the characteristics of malignancy. Moreover, the Mann–Whitney test shows a statistical difference in terms of the size and age between benign and malignant nodules due to (P <.001). More importantly, the selected characteristics are more critical than the remaining characteristics for thyroid nodule diagnosis on the training dataset (e.g., see Fig. 5). The selected characteristics are marked by bold. Reference categories for each of US characteristics are those with the lowest malignancy rate in that our main purpose is to select active predictors associated with malignancy.

**Table 2:** NPV, PPV, SEN, SPE and AUC values for each of US characteristic in disease nodules

Characteristic	Benign	Malignant	NPV	PPV	SEN	SPE	AUC
Shape							
*AP*/*T*≥1	34(4.7%)	684(95.3%)	29.3%	95.3%	57.0%	86.3%	0.716
*AP*/*T*<1	214(29.3%)	516(70.7%)	Reference				
Margin							
Regular	174(49.2%)	180(50.8%)	Reference				
Irregular	74(6.8%)	1020(93.2%)	49.2%	93.2%	85.0%	70.2%	0.776
Border							
Unclear	55(6.3%)	819(93.7%)	33.6%	93.7%	68.2%	77.8%	0.730
Clear	193(33.6%)	381(66.4%)	Reference				
Hackly border							
Present	22(3.0%)	744(97%)	33.1%	97.1%	62.0%	91.1%	0.766
Absent	226 (33.1%)	456(66.9%)	Reference				
Component							
Solid	108(8.5%)	1157(91.5%)	76.5%	91.5%	96.4%	56.5%	0.764
Mixed	140(76.5%)	43(23.5%)	Reference				
Calcifications							
Macro-calcifications	30(18.0%)	137(82.0%)	17.0%	82.0%	11.4%	87.9%	0.497
Micro-calcifications	78(9.9%)	711(90.1%)	25.8%	90.1%	59.2%	68.5%	0.639
No calcifications	140(28.5%)	352(71.5%)	Reference				
Halo							
Present	209(15.7%)	1123(84.3%)	33.6%	84.3%	93.6%	15.7%	0.547
Absent	39(33.6%)	77(66.4%)	Reference				
Attenuation							
Present	12(5.5%)	208(94.5%)	19.2%	94.5%	17.3%	95.2%	0.562
Absent	236(19.2%)	992(80.8%)	Reference				
Lateral shadow							
Present	5(8.8%)	52(91.2%)	17.5%	91.2%	4.3%	98.0%	0.512
Absent	243(17.5%)	1148(82.5%)	Reference				
Blood flow degree							
Low	49(10.5%)	417(89.5%)	20.3%	89.5%	34.8%	80.2%	0.575
Medium	116(22.7%)	396(77.3%)	Reference				
High	83(17.7%)	387(82.3%)	16.9%	82.3%	32.2%	66.5%	0.494
Vascularity							
Peripheral	78(23.8%)	250(76.2%)	Reference				
Mixed	69(23.2%)	228(76.8%)	15.6%	76.8%	19.0%	72.2%	0.456
Central	101(12.3%)	722(87.7%)	23.5%	87.7%	60.2%	59.3%	0.597
Echogenicity							
Hypoechoic	117(11.3%)	920(88.7%)	31.9%	88.7%	76.7%	52.8%	0.647
Hyperechoic	131(31.9%)	280(68.1%)	Reference				

### Performance of the predictive model

We utilize the class probabilities to predict the risk of malignancy for each of the nodules on the basis of the classifiers obtained from the training dataset. A nodule is predicted as malignancy if the class probability has a higher value than the given cutoff (the optimal cutoff point on the AUC closest to (0,1)). To measure the performance of the four classifiers, we use six metrics: AUC, SEN, F1 score, SPE, PPV, NPV, which are the averages calculated with 100 repetitions. As is shown in Table 3, the LR (i.e., LR model with stepwise selection procedure) show the highest AUC (i.e., 0.965) regardless of the validating and testing datasets, the RF produces the highest SEN (i.e., 89.2%) for the validating dataset and the second highest SEN (i.e., 88.3%) for the testing dataset, the ADAB has the highest F1 (i.e., 0.73) for the validating dataset, the NET leads to the highest F1 (i.e., 74.6%) for the testing dataset, the LOG produces the highest SPE (i.e., 96.0%) regardless of the validating and testing datasets, the SVM shows the highest PPV (i.e., 98.1%) for the validating dataset, and the ADAB yields the highest PPV (i.e., 98.5%) for the testing dataset and has the highest NPV (i.e., 62.9%) for the validating dataset. These observations show the evidence that none of ten classifiers performs best at all metrics when we only use a cutoff of the class probability to differentiate between benign and malignant cases.

**Table 3:** Comparison of classification performance of machine learning methods on validating and testing datasets

Validating data					Testing data								
	AUC	SEN	F1	SPE	PPV	NPV		AUC	SEN	F1	SPE	PPV	NPV
RF	0.960	0.892	0.701	0.820	0.960	0.612		0.958	0.883	0.721	0.880	0.972	0.611
LR	0.965	0.875	0.720	0.900	0.977	0.600		0.965	0.875	0.720	0.900	0.977	0.600
SVM	0.952	0.862	0.713	0.920	0.981	0.582		0.962	0.896	0.740	0.900	0.977	0.643
NET	0.965	0.879	0.726	0.900	0.977	0.608		0.964	0.879	0.746	0.920	0.981	0.613
ELM	0.952	0.833	0.657	0.880	0.971	0.524		0.952	0.833	0.657	0.880	0.971	0.524
KNN	0.926	0.825	0.657	0.900	0.975	0.517		0.926	0.825	0.657	0.900	0.975	0.517
NB	0.940	0.871	0.672	0.820	0.959	0.569		0.940	0.871	0.672	0.820	0.959	0.569
ADAB	0.955	0.891	0.733	0.880	0.973	0.629		0.958	0.855	0.712	0.940	0.985	0.574
LOG	0.964	0.846	0.711	0.960	0.970	0.565		0.956	0.846	0.711	0.960	0.970	0.565
LDA	0.954	0.850	0.687	0.900	0.976	0.556		0.954	0.850	0.687	0.900	0.976	0.556

### The scoring system of thyroid nodules

**Fig. 6:**
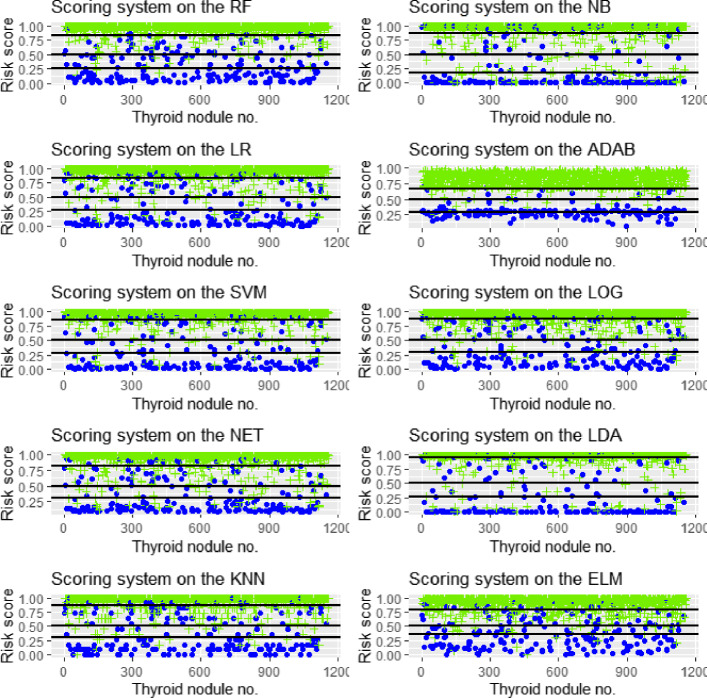
The risk score of malignancy for each thyroid nodule, as calculated by statistics methods respectively. The green crossings represent malignant nodules and blue dots benign nodules

Only using the cutoff of the class probability to differentiate thyroid cancer may result in an increase in misdiagnosis or missed diagnosis due to the considerable overlapping of the US characteristics for benign and malignant nodules [6, 24, 25]. Categorizing nodules and stratifying their risks of malignancy according to the risk score (class probability) may be one of the most efficient approaches to solve this problem. The greater risk score suggests a higher malignant risk. Figure 6 displays the sores for each of nodules for ten classifiers on the training and validating datasets. It is observed that most of the scores associated with malignant nodules are greater than those associated with benign nodules. Figure 6 shows the risk scores of benign and malignant nodules much more overlapped for LR, SVM, NET, ELM at the bottom of the band, which indicate that these classifiers score lowly for some malignant nodules; thus a true malignant nodule may be incorrectly classified as a benign one.

**Fig. 7:**
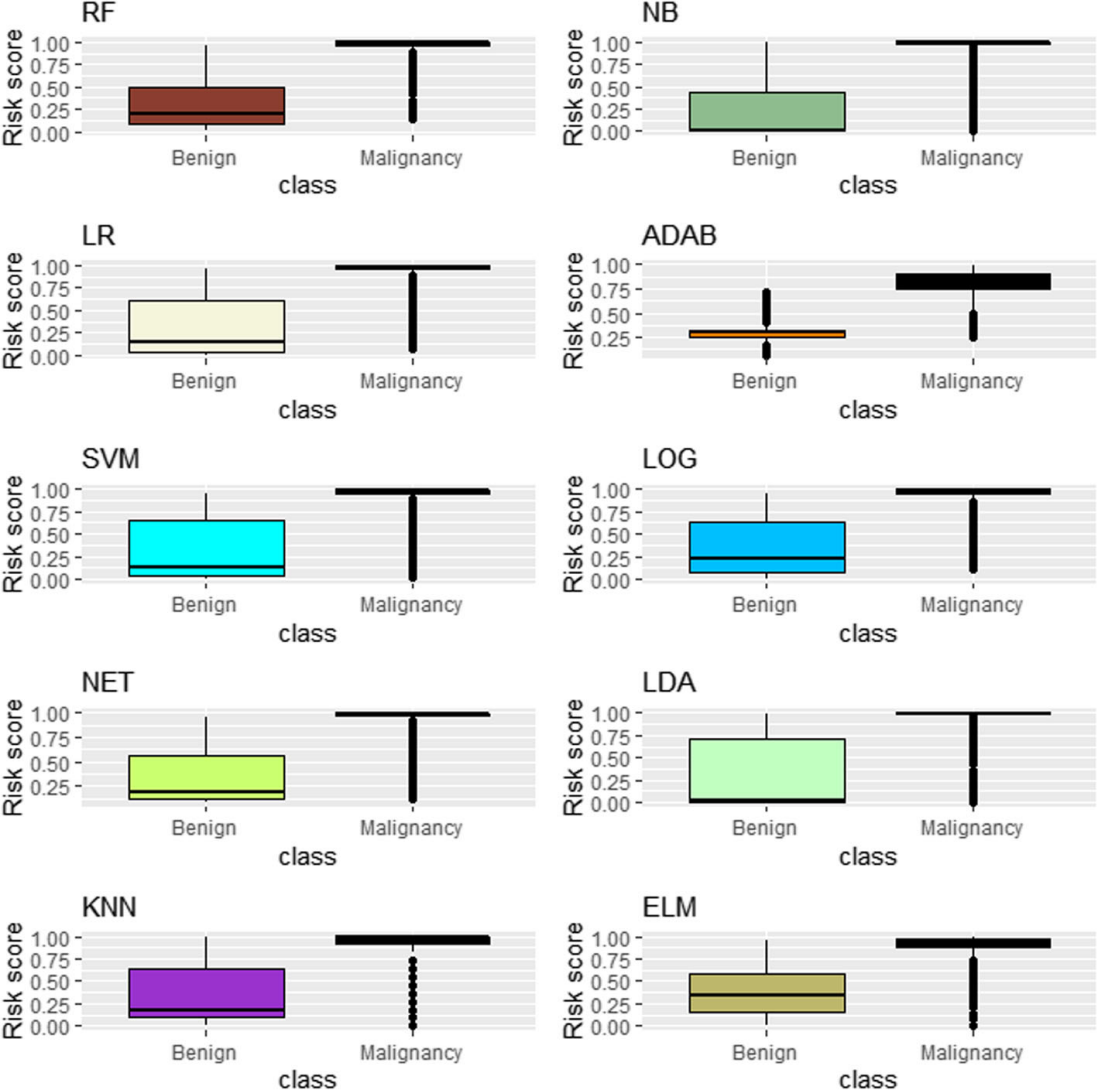
The box bar graphs show the risk score of malignancy for benign and malignant nodules

For benign nodules, 75% of risk scores is less than 0.497 using the RF. In contrast, 75% of risk scores is less than 0.617 using the LR, 0.644 using the SVM, 0.561 using the NET, 0.588 using the ELM, 0.636 using the KNN, 0.437 using the NB, 0.313 using the ADAB, 0.635 using the LOG and 0.712 using the LDA (e.g., see Fig. 7). Meanwhile, for malignant nodules, the lowest *S*_RF_,*S*_LR_,*S*_SVM_,*S*_NET_,*S*_ELM_*S*_KNN_,*S*_NB_,*S*_ADAB_,*S*_LOG_ and *S*_LDA_ are 0.152, 0.065, 0.025, 0.128, 0.002, 0.000, 0.000, 0.221, 0.105 and 0.006, respectively.

We establish a classification system for thyroid nodules (e.g., see Table 4) in terms of risk scores. For training and validating datasets, 1158 (malignant case=960, benign case=198) thyroid nodules are classified into four categories via the risk scores: benign category, in which the nodule has a score less than the 95% confidence lower limit *l*_*c*_ of its mean calculated using the bootstrap percentile method for 1000 bootstrap replications; low suspicion category, in which the nodule has a score ranged from *l*_*c*_ to 0.5; intermediate suspicion category that includes the nodules with scores ranged from 0.5 to the cutoff *h*_*c*_ on the AUC closest to (0,1); high suspicion of malignancy category, in which the nodule has a score greater than *h*_*c*_.

**Table 4:** Risk scoring system of thyroid nodules on the training and validating dataset

	Benign		Low suspicion		Intermediate suspicion		High suspicion	
RF	*S*_*RF*_<*l*_*c*_(0.265)		*l*_*c*_≤*S*_*RF*_<0.5		0.5≤*S*_*RF*_<*h*_*c*_(0.784)		*S*_*RF*_≥*h*_*c*_	
	benign	malignancy	benign	malignancy	benign	malignancy	benign	malignancy
	110	4	41	11	35	47	12	898
	96.5%	3.5%	78.8%	21.2%	42.7%	57.3%	1.3%	98.7%
LR	*S*_*LR*_<*l*_*c*_(0.267)		*l*_*c*_≤*S*_*LR*_<0.5		0.5≤*S*_*LR*_<*h*_*c*_(0.838)		*S*_*LR*_≥*h*_*c*_	
	benign	malignancy	benign	malignancy	benign	malignancy	benign	malignancy
	123	17	13	16	42	81	20	846
	87.9%	12.1%	44.8%	55.2%	34.1%	65.9%	2.3%	97.7%
SVM	*S*_*SVM*_<*l*_*c*_(0.277)		*l*_*c*_≤*S*_*SVM*_<0.5		0.5≤*S*_*SVM*_<*h*_*c*_(0.850)		*S*_*SVM*_≥*h*_*c*_	
	benign	malignancy	benign	malignancy	benign	malignancy	benign	malignancy
	115	16	23	16	35	71	25	857
	87.8%	12.2%	59.0%	41.0%	33.0%	67.0%	2.8%	97.2%
NET	*S*_*NET*_<*l*_*c*_(0.300)		*l*_*c*_≤*S*_*NET*_<0.5		0.5≤*S*_*NET*_<*h*_*c*_(0.820)		*S*_*NET*_≥*h*_*c*_	
	benign	malignancy	benign	malignancy	benign	malignancy	benign	malignancy
	125	19	14	20	37	61	22	860
	86.8%	13.2%	41.2%	58.8%	34.8%	65.2%	2.3%	97.7%
ELM	*S*_*ELM*_<*l*_*c*_(0.352)		*l*_*c*_≤*S*_*ELM*_<0.5		0.5≤*S*_*ELM*_<*h*_*c*_(0.783)		*S*_*ELM*_≥*h*_*c*_	
	benign	malignancy	benign	malignancy	benign	malignancy	benign	malignancy
	103	7	28	18	46	113	21	822
	93.6%	6.4%	60.9%	39.1%	29.0%	71.0%	2.5%	97.5%
KNN	*S*_*KNN*_<*l*_*c*_(0.305)		*l*_*c*_≤*S*_*KNN*_<0.5		0.5≤*S*_*KNN*_<*h*_*c*_(0.864)		*S*_*KNN*_≥*h*_*c*_	
	benign	malignancy	benign	malignancy	benign	malignancy	benign	malignancy
	117	13	15	16	49	114	17	817
	90.0%	10.0%	48.4%	51.6%	30.1%	69.9%	2.0%	98.0%
NB	*S*_*NB*_<*l*_*c*_(0.188)		*l*_*c*_≤*S*_*NB*_<0.5		0.5≤*S*_*NB*_<*h*_*c*_(0.869)		*S*_*NB*_≥*h*_*c*_	
	benign	malignancy	benign	malignancy	benign	malignancy	benign	malignancy
	138	45	15	25	18	67	27	823
	75.4%	24.6%	37.5%	62.5%	21.2%	78.8%	3.2%	96.8%
ADAB	*S*_*ADAB*_<*l*_*c*_(0.286)		*l*_*c*_≤*S*_*ADAB*_<0.5		0.5≤*S*_*ADAB*_<*h*_*c*_(0.614)		*S*_*ADAB*_≥*h*_*c*_	
	benign	malignancy	benign	malignancy	benign	malignancy	benign	malignancy
	87	2	98	12	7	12	6	934
	97.8%	2.2%	89.1%	10.9%	36.9%	63.1%	0.6%	99.4%
LOG	*S*_*LOG*_<*l*_*c*_(0.317)		*l*_*c*_≤*S*_*LOG*_<0.5		0.5≤*S*_*LOG*_<*h*_*c*_(0.820)		*S*_*LOG*_≥*h*_*c*_	
	benign	malignancy	benign	malignancy	benign	malignancy	benign	malignancy
	113	13	19	12	52	118	14	817
	89.7%	10.3%	61.3%	38.7%	30.6%	69.4%	1.7%	98.3%
LDA	*S*_*LDA*_<*l*_*c*_(0.256)		*l*_*c*_≤*S*_*LDA*_<0.5		0.5≤*S*_*LDA*_<*h*_*c*_(0.958)		*S*_*LDA*_≥*h*_*c*_	
	benign	malignancy	benign	malignancy	benign	malignancy	benign	malignancy
	120	27	18	10	41	100	19	823
	81.6%	18.4%	64.3%	35.7%	29.1%	70.9%	2.3%	97.7%
Management	After a 6-month		After 3-month sonographic				FNA biopsy	
of thyroid	sonographic		follow-up or Fine Needle		FNA biopsy		or surgical	
nodules	follow-up		Aspiration (FNA) biopsy				treatment	

As is shown in Table 4, using the RF, the malignancy rate achieves the lower value in the benign category and the higher value in the high suspicion category; while for ADAB method, although it can get the lowest malignancy rate in the benign categroy and the highest malignancy rate in the high suspicion category, but its computation is time-consuming. We also recommend guidelines for the management of thyroid nodules according to their categories. The risk scoring system on the basis of RF is superior to those of other methods in diagnosing thyroid cancer in terms of malignancy rates of 3.5%, 21.2%, 57.3%, and 98.7% in benign category, low suspicion category, intermediate suspicion category, and high suspicion of malignancy category, respectively.

### Final validation

To avoid the over-fitting of the classifiers and test the reliability of the risk scoring system using the RF, we conduct the final validation on the testing dataset. The results are given in Table 5. Among the considered ten classifiers, the RF yields malignancy rates of 0%, 14.3%, 58.1% and 96.1% in benign category, low suspicion category, intermediate suspicion category, and high suspicion of malignancy category, respectively, compared with 4.2%, 37.5%, 63.0%, and 96.9% for the LR, 14.8%, 37.5%, 58.3%, and 96.8% for the SVM, 3.7%, 35.3%, 69.6%, and 97.3% for the NET, 4.3%, 50.0%, 59.1%, and 97.7% for the ELM, 8%, 11.1%, 73.3%, and 96.7% for the KNN, 22%, 71.4%, 67.9%, and 96.7% for the NB, 0%, 26.7%, 82.4%, and 93.6% for the ADAB, 5.3%, 23.5%, 64.3%, and 98.1% for the LOG, 6.9%, 33.3%, 64.9%, and 97.2% for the LDA, which show that RF method outperforms other nine methods.

**Table 5:** Risk scoring system of thyroid nodules on the testing dataset

	Benign		Low suspicion		Intermediate suspicion		High suspicion	
RF	*S*_*RF*_<*l*_*c*_(0.265)		*l*_*c*_≤*S*_*RF*_<0.5		0.5≤*S*_*RF*_<*h*_*c*_(0.784)		*S*_*RF*_≥*h*_*c*_	
	benign	malignancy	benign	malignancy	benign	malignancy	benign	malignancy
	16	0	12	2	13	18	9	220
	100%	0%	75.7%	14.3%	41.9%	58.1%	3.9%	96.1%
LR	*S*_*LR*_<*l*_*c*_(0.267)		*l*_*c*_≤*S*_*LR*_<0.5		0.5≤*S*_*LR*_<*h*_*c*_(0.838)		*S*_*LR*_≥*h*_*c*_	
	benign	malignancy	benign	malignancy	benign	malignancy	benign	malignancy
	23	1	10	6	10	17	7	216
	95.8%	4.2%	62.5%	37.5%	37.0%	63.0%	3.1%	96.9%
SVM	*S*_*SVM*_<*l*_*c*_(0.277)		*l*_*c*_≤*S*_*SVM*_<0.5		0.5≤*S*_*SVM*_<*h*_*c*_(0.850)		*S*_*SVM*_≥*h*_*c*_	
	benign	malignancy	benign	malignancy	benign	malignancy	benign	malignancy
	23	4	5	3	15	21	7	212
	85.2%	14.8%	62.5%	37.5%	41.7%	58.3%	3.2%	96.8%
NET	*S*_*NET*_<*l*_*c*_(0.300)		*l*_*c*_≤*S*_*NET*_<0.5		0.5≤*S*_*NN*_<*h*_*c*_(0.820)		*S*_*NET*_≥*h*_*c*_	
	benign	malignancy	benign	malignancy	benign	malignancy	benign	malignancy
	26	1	11	6	7	16	7	217
	96.3%	3.7%	64.7%	35.3%	30.4%	69.6%	3.1%	96.9%
ELM	*S*_*ELM*_<*l*_*c*_(0.352)		*l*_*c*_≤*S*_*ELM*_<0.5		0.5≤*S*_*ELM*_<*h*_*c*_(0.783)		*S*_*ELM*_≥*h*_*c*_	
	benign	malignancy	benign	malignancy	benign	malignancy	benign	malignancy
	22	1	5	5	18	26	5	208
	95.7%	4.3%	50.0%	50.0%	40.9%	59.1%	2.3%	97.7%
KNN	*S*_*KNN*_<*l*_*c*_(0.305)		*l*_*c*_≤*S*_*KNN*_<0.5		0.5≤*S*_*KNN*_<*h*_*c*_(0.864)		*S*_*KNN*_≥*h*_*c*_	
	benign	malignancy	benign	malignancy	benign	malignancy	benign	malignancy
	23l	2	8	1	12	33	7	204
	92.0%	8.0%	88.9%	11.1%	26.7%	73.3%	3.3%	96.7%
NB	*S*_*NB*_<*l*_*c*_(0.188)		*l*_*c*_≤*S*_*NB*_<0.5		0.5≤*S*_*NB*_<*h*_*c*_(0.869)		*S*_*NB*_≥*h*_*c*_	
	benign	malignancy	benign	malignancy	benign	malignancy	benign	malignancy
	32	9	2	5	9	19	7	207
	78.0%	22.0%	28.6%	71.4%	32.1%	67.9%	3.3%	96.7%
ADAB	*S*_*ADA*_<*l*_*c*_(0.286)		*l*_*c*_≤*S*_*ADA*_<0.5		0.5≤*S*_*ADA*_<*h*_*c*_(0.614)		*S*_*ADA*_≥*h*_*c*_	
	benign	malignancy	benign	malignancy	benign	malignancy	benign	malignancy
	10	0	22	8	3	14	15	218
	100.0%	0.0%	73.3%	26.7%	17.6%	82.4%	6.4%	93.6%
LOG	*S*_*LOG*_<*l*_*c*_(0.317)		*l*_*c*_≤*S*_*LOG*_<0.5		0.5≤*S*_*LOG*_<*h*_*c*_(0.820)		*S*_*LOG*_≥*h*_*c*_	
	benign	malignancy	benign	malignancy	benign	malignancy	benign	malignancy
	18	1	13	4	15	27	7	205
	94.7%	5.3%	76.5%	23.5%	35.7%	64.3%	3.3%	96.7%
LDA	*S*_*LDA*_<*l*_*c*_(0.256)		*l*_*c*_≤*S*_*LDA*_<0.5		0.5≤*S*_*LDA*_<*h*_*c*_(0.958)		*S*_*LDA*_≥*h*_*c*_	
	benign	malignancy	benign	malignancy	benign	malignancy	benign	malignancy
	27	2	4	2	13	24	6	212
	93.1%	6.9%	66.7%	33.3%	35.1%	64.9%	2.8%	97.2%
Management	After a 6-month		After 3-month				FNA biopsy	
of thyroid	sonographic		sonographic follow-up		FNA biopsy		or surgical	
nodules	follow-up		or FNA biopsy				treatment	

### Abnormal nodules

In the abnormal nodules, there is considerable overlap between the characteristics of malignant and benign nodules. Fifty-five (55.6%) of 99 benign nodules has AP/T≥1; 69 (69.7%) has hackly border; 61 (61.6%) contains micro-calcifications; and 100% is solid. In contrast, among 11 malignant nodules, only one (9.1%) has AP/T≥1, hackly border, solid component, and micro-calcifications, respectively, which have significant association with malignancy. The values of SEN, SPE, PPV, and AUC for each of US characteristics for the abnormal nodules are lower than those for the disease nodules.

When the abnormal nodules are added to the disease nodules, the performance metrics of all the US characteristics decrease except for NPV (e.g., see Table 6), and the performance metrics of four classifiers (e.g., see Table 7) also decrease. For example, Table 7 shows that the LR and LDA methods show the highest AUC (i.e., 0.820), the NB method produces the highest SEN (i.e., 85.1%) and NPV (i.e., 55.0%), the LR method has the highest F1 (i.e., 0.611). While the NET method produces the highest SPE (i.e., 74.3%) and PPV (i.e., 91.4%). At the same time, the RF method yields better results than other methods in terms of risk scores and risk scoring system (e.g., see Tables 8 and 9).

**Table 6:** NPV, PPV, SEN, SPE and AUC values for each of US characteristic in overall nodules

Characteristic	Benign	Malignant	NPV	PPV	SEN	SPE	AUC
Shape							
*AP*/*T*≥1	89	685	32.9%	88.5%	56.6%	74.4%	0.655
*AP*/*T*<1	258	526			Reference		
Margin							
Regular	180	189			Reference		
Irregular	167	1022	48.8%	86.0%	84.4%	51.9%	0.681
Border							
Unclear	137	820	34.9%	85.7%	67.7%	60.5%	0.641
Clear	210	391			Reference		
Hackly border							
Present	91	745	35.5%	89.1%	61.5%	73.8%	0.676
Absent	256	466			Reference		
Component							
Solid	207	1158	72.5%	84.8%	95.6%	40.3%	0.680
Mixed	140	53			Reference		
Calcifications							
Macro-calcifications	46	137	21.9%	74.9%	11.3%	86.7%	0.490
Micro-calcifications	139	712	29.4%	83.7%	58.8%	59.9%	0.594
No calcifications	162	362			Reference		
Halo							
Present	303	1132	35.8%	78.9%	93.5%	12.7%	0.531
Absent	44	79			Reference		
Attenuation							
Present	33	208	23.8%	86.3%	17.2%	90.5%	0.538
Absent	314	1003			Reference		
Lateral shadow							
Present	9	52	22.6%	85.2%	4.3%	97.4%	0.509
Absent	338	1159			Reference		
Blood flow degree							
Low	80	417	25.2%	83.9%	34.4%	76.9%	0.557
Medium	152	402			Reference		
High	115	392	22.1%	77.3%	32.4%	66.9%	0.496
Vascularity							
Peripheral	103	255			Reference		
Mixed	88	231	20.9%	72.4%	19.1%	74.6%	0.469
Central	156	725	28.2%	82.3%	59.9%	55.0%	0.575
Echogenicity							
Hypoechoic	198	925	34.3%	82.4%	76.4%	42.9%	0.597
Hyperechoic	149	286			Reference

**Table 7:** Comparison of classification performance of machine learning methods on validating dataset of overall nodules

	AUC	SEN	F1	SPE	PPV	NPV
RF	0.798	0.822	0.588	0.672	0.896	0.522
LR	0.820	0.810	0.611	0.729	0.912	0.526
SVM	0.781	0.777	0.558	0.685	0.895	0.470
NET	0.818	0.789	0.601	0.743	0.914	0.505
ELM	0.811	0.806	0.573	0.671	0.894	0.500
KNN	0.738	0.707	0.492	0.657	0.877	0.393
NB	0.796	0.851	0.587	0.629	0.888	0.550
ADAB	0.748	0.777	0.506	0.599	0.870	0.437
LOG	0.811	0.810	0.610	0.729	0.912	0.526
LDA	0.820	0.793	0.588	0.714	0.906	0.500

**Table 8:** Risk scoring system of thyroid nodules on training and validating dataset in overall nodules

RF	*S*_*RF*_<*l*_*c*_(0.247)		*l*_*c*_≤*S*_*RF*_<0.5		0.5≤*S*_*RF*_<*h*_*c*_(0.714)		*S*_*RF*_≥*h*_*c*_	
	Benign		Low suspicion		Intermediate suspicion		High suspicion	
	benign	malignancy	benign	malignancy	benign	malignancy	benign	malignancy
	145	6	91	14	21	37	21	912
	96.0%	4.00%	86.7%	13.3%	36.2%	63.8%	2.2%	97.8%
LR	*S*_*LR*_<*l*_*c*_(0.470)		*l*_*c*_≤*S*_*LR*_<0.5		0.5≤*S*_*LR*_<*h*_*c*_(0.796)		*S*_*LR*_≥*h*_*c*_	
	benign	malignancy	benign	malignancy	benign	malignancy	benign	malignancy
	130	35	5	8	74	165	69	761
	78.8%	21.2%	38.5%	61.5%	31.0%	69.0%	8.3%	91.7%
SVM	*S*_*SVM*_<*l*_*c*_(0.467)		*l*_*c*_≤*S*_*SVM*_<0.5		0.5≤*S*_*SVM*_<*h*_*c*_(0.863)		*S*_*SVM*_≥*h*_*c*_	
	benign	malignancy	benign	malignancy	benign	malignancy	benign	malignancy
	122	24	4	2	81	166	68	777
	83.6%	16.4%	66.7%	33.3%	33.6%	66.4%	8.0%	92.0%
NET	*S*_*NET*_<*l*_*c*_(0.465)		*l*_*c*_≤*S*_*NET*_<0.5		0.5≤*S*_*NET*_<*h*_*c*_(0.840)		*S*_*NET*_≥*h*_*c*_	
	benign	malignancy	benign	malignancy	benign	malignancy	benign	malignancy
	135	32	3	3	66	169	74	765
	80.8%	19.2%	50.0%	50.0%	29.1%	71.9%	8.8%	91.2%
ELM	*S*_*ELM*_<*l*_*c*_(0.409)		*l*_*c*_≤*S*_*ELM*_<0.5		0.5≤*S*_*ELM*_<*h*_*c*_(0.779)		*S*_*ELM*_≥*h*_*c*_	
	benign	malignancy	benign	malignancy	benign	malignancy	benign	malignancy
	120	27	0	0	89	182	70	760
	81.6%	18.4%	—	—	32.8%	67.2%	8.4%	91.6%
KNN	*S*_*KNN*_<*l*_*c*_(0.466)		*l*_*c*_≤*S*_*KNN*_<0.5		0.5≤*S*_*KNN*_<*h*_*c*_(0.833)		*S*_*KNN*_≥*h*_*c*_	
	benign	malignancy	benign	malignancy	benign	malignancy	benign	malignancy
	125	31	0	0	96	247	57	691
	80.1%	19.9%	—	—	28.0%	72.0%	7.6%	92.4%
NB	*S*_*NB*_<*l*_*c*_(0.391)		*l*_*c*_≤*S*_*NB*_<0.5		0.5≤*S*_*NB*_<*h*_*c*_(0.836)		*S*_*NB*_≥*h*_*c*_	
	benign	malignancy	benign	malignancy	benign	malignancy	benign	malignancy
	148	72	9	14	27	90	94	793
	67.3%	32.7%	39.1%	60.9%	23.1%	76.9%	10.6%	89.4%
ADAB	*S*_*ADAB*_<*l*_*c*_(0.406)		*l*_*c*_≤*S*_*ADAB*_<0.5		0.5≤*S*_*ADAB*_<*h*_*c*_(0.560)		*S*_*ADAB*_≥*h*_*c*_	
	benign	malignancy	benign	malignancy	benign	malignancy	benign	malignancy
	135	9	100	24	15	36	28	900
	93.8%	6.2%	80.6%	19.4%	29.4%	70.6%	3.0%	97.0%
LOG	*S*_*LOG*_<*l*_*c*_(0.498)		*l*_*c*_≤*S*_*LOG*_<0.5		0.5≤*S*_*LOG*_<*h*_*c*_(0.802)		*S*_*LOG*_≥*h*_*c*_	
	benign	malignancy	benign	malignancy	benign	malignancy	benign	malignancy
	127	38	0	0	80	167	71	764
	77.0%	23.0%	—	—	32.4%	67.6%	8.5%	91.5%
LDA	*S*_*LDA*_<*l*_*c*_(0.464)		*l*_*c*_≤*S*_*LDA*_<0.5		0.5≤*S*_*LDA*_<*h*_*c*_(0.879)		*S*_*LDA*_≥*h*_*c*_	
	benign	malignancy	benign	malignancy	benign	malignancy	benign	malignancy
	130	39	3	8	73	172	72	750
	76.9%	23.1%	27.3%	72.7%	29.8%	70.2%	8.8%	91.2%
Management	After a 6-month		After 3-month				FNA biopsy	
of thyroid	sonographic		sonographic follow-up		FNA biopsy		or surgical	
nodules	follow-up		or FNA biopsy				treatment

**Table 9:** Risk scoring system of thyroid nodules on testing dataset in overall nodules

RF	*S*_*RF*_<*l*_*c*_(0.439)		*l*_*c*_≤*S*_*RF*_<0.5		0.5≤*S*_*RF*_<*h*_*c*_(0.849)		*S*_*RF*_≥*h*_*c*_	
	Benign		Low suspicion		Intermediate suspicion		High suspicion	
	benign	malignancy	benign	malignancy	benign	malignancy	benign	malignancy
	34	5	3	4	15	54	17	179
	87.2%	12.8%	42.9%	57.1%	21.7%	78.3%	8.7%	91.3%
LR	*S*_*LR*_<*l*_*c*_(0.486)		*l*_*c*_≤*S*_*LR*_<0.5		0.5≤*S*_*LR*_<*h*_*c*_(0.817)		*S*_*LR*_≥*h*_*c*_	
	benign	malignancy	benign	malignancy	benign	malignancy	benign	malignancy
	37	15	1	1	14	54	17	172
	71.2%	28.8%	50.0%	50.0%	20.6%	79.4%	9.0%	91.0%
SVM	*S*_*SVM*_<*l*_*c*_(0.467)		*l*_*c*_≤*S*_*SVM*_<0.5		0.5≤*S*_*SVM*_<*h*_*c*_(0.863)		*S*_*SVM*_≥*h*_*c*_	
	benign	malignancy	benign	malignancy	benign	malignancy	benign	malignancy
	37	13	1	1	17	60	15	168
	74.0%	26.0%	50.0%	50.0%	22.1%	77.9%	8.2%	91.8%
NET	*S*_*NET*_<*l*_*c*_(0.487)		*l*_*c*_≤*S*_*NET*_<0.5		0.5≤*S*_*NET*_<*h*_*c*_(0.854)		*S*_*NET*_≥*h*_*c*_	
	benign	malignancy	benign	malignancy	benign	malignancy	benign	malignancy
	37	16	0	1	14	54	18	171
	69.8%	30.2%	0.0%	100.0%	20.6%	79.4%	9.5%	90.5%
ELM	*S*_*ELM*_<*l*_*c*_(0.409)		*l*_*c*_≤*S*_*ELM*_<0.5		0.5≤*S*_*ELM*_<*h*_*c*_(0.779)		*S*_*ELM*_≥*h*_*c*_	
	benign	malignancy	benign	malignancy	benign	malignancy	benign	malignancy
	35	11	0	0	20	61	14	172
	76.1%	23.9%	—	—	24.7%	75.3%	7.5%	92.5%
KNN	*S*_*KNN*_<*l*_*c*_(0.466)		*l*_*c*_≤*S*_*KNN*_<0.5		0.5≤*S*_*KNN*_<*h*_*c*_(0.833)		*S*_*KNN*_≥*h*_*c*_	
	benign	malignancy	benign	malignancy	benign	malignancy	benign	malignancy
	39	12	0	0	14	70	16	160
	76.5%	23.5%	—	—	16.7%	83.3%	9.1%	90.9%
NB	*S*_*NB*_<*l*_*c*_(0.391)		*l*_*c*_≤*S*_*NB*_<0.5		0.5≤*S*_*NB*_<*h*_*c*_(0.836)		*S*_*NB*_≥*h*_*c*_	
	benign	malignancy	benign	malignancy	benign	malignancy	benign	malignancy
	44	32	1	5	4	23	20	182
	57.9%	42.1%	16.7%	83.3%	14.8%	85.2%	9.9%	90.1%
ADAB	*S*_*ADAB*_<*l*_*c*_(0.406)		*l*_*c*_≤*S*_*ADAB*_<0.5		0.5≤*S*_*ADAB*_<*h*_*c*_(0.560)		*S*_*ADAB*_≥*h*_*c*_	
	benign	malignancy	benign	malignancy	benign	malignancy	benign	malignancy
	20	9	21	22	10	24	18	187
	69.0%	31.0%	48.8%	51.2%	29.4%	70.6%	8.8%	91.2%
LOG	*S*_*LOG*_<*l*_*c*_(0.498)		*l*_*c*_≤*S*_*LOG*_<0.5		0.5≤*S*_*LOG*_<*h*_*c*_(0.802)		*S*_*LOG*_≥*h*_*c*_	
	benign	malignancy	benign	malignancy	benign	malignancy	benign	malignancy
	34	17	0	0	17	47	18	178
	66.7%	33.3%	—	—	26.6%	73.4%	9.2%	90.8%
LDA	*S*_*LDA*_<*l*_*c*_(0.464)		*l*_*c*_≤*S*_*LDA*_<0.5		0.5≤*S*_*LDA*_<*h*_*c*_(0.879)		*S*_*LDA*_≥*h*_*c*_	
	benign	malignancy	benign	malignancy	benign	malignancy	benign	malignancy
	37	15	1	0	13	54	18	173
	71.2%	28.8%	100.0%	0%	19.4%	80.6%	9.4%	90.6%
Management	After a 6-month		After 3-month				FNA biopsy	
of thyroid	sonographic		sonographic follow-up		FNA biopsy		or surgical	
nodules	follow-up		or FNA biopsy				treatment	

## Discussion

From LLR, the US characteristics: tumor size, AP/T, solid component, micro-calcifications, hackly border, hypoechogenic area, present halo, unclear border, irregular shape, and central vascularity were showed significant association with malignancy. In fact, previous studies have shown that the presence of AP/T, solid component, micro-calcifications, irregular shape were consistently associated with a higher risk of malignancy [8]; absent halo and vascular pattern can be suggestive of malignancy [4]; tumor size and hackly border were risk factor for detecting malignanct nodules [10, 26]. Results obtained with the LLR method were compared with those of the management guidelines [10] and many previous studies [3, 8, 26], in which a solid hypoechoic nodule or solid hypoechoic component of a partially cystic nodule has the following one or many characteristics: hackly border, micro-calcifications, AP/T>1, high suspicion US pattern. The comparison indicates the effectiveness of the LLR method for selecting active features. Incorporating these characteristics as predictors has relatively higher SEN, AUC, SPE, NPV, and PPV values than those only using one of the characteristics. Consequently, a combination of highly correlated characteristics can indeed improve the performance of the prediction of malignancy-risk compared with the usage of a single characteristic, which is consistent with that no single US feature on its own can reliably differentiate malignant nodules from benign ones [12].

Our proposed hybrid method (i.e., incorporating LLR and RF) can not only select important US features via LASSO but also obtain risk score via the LR model with the selected predictors, which is a basic information for classification and leads to a more effective and objective diagnosis than conventional classifiers discussed in Zhang et al. [12]. Although Zhang et al. [12] compared the performance of conventional classifiers with that of RF method and recommended the uage of RF method, but they did not provide a quantitative approach to assess the risk of the malignant nodule and consider to calculate the risk score of thyroid nodules, leading to unknown information on the level of risk for the classifier. At the same time, statistical results show that our proposed hybrid classifier outperforms other classifiers such as LR, SVM, NET, ELM, NB, ADA, LOG and LAD in terms of their corresponding malignancy rates, which implies that incorporating LR model with the incorporated predictors and RF method can improve the performance of the prediction of malignancy. In particular, our proposed method behaves better than the widely used RF method in terms of risk score sytem in that we utilize the optimal cutoff point to replace the default cutoff point in implementing RF algorithm. Although extreme learning machine (ELM) has been explored to discriminate malignant and benign thyroid nodules based on the sonographic features in ultrasound images [22], but then did’t compare with other methods. In addition, in the previous studies (e.g., see [7, 12, 22, 24]), thyroid nodules were classified into two kinds: benign and malign based on the US characteristics together with the default cutoff of class probability (i.e., 0.5), which may result in an increase in misdiagnosis or missed diagnosis due to the considerable US characteristics common to benign and malignant nodules. In tihs study, we categorized nodules into the following four categories: benign, low suspicion, intermediate suspicion, high suspicion according to the risk score of thyroid nodule and 2015 American Thyroid Association management guidelines [10].

US characteristics, which are suggestiveness of thyroid malignancy, should be indication for Fine Needle Aspiration (FNA) biopsy and even further treatment such as surgery. However, different levels of clinical experience and description of US findings might cause diverse diagnostic accuracies. Thus, there is a significant demand to establish some objective criteria to select nodules for FNA biopsy or surgery to minimize costs. In our study, we scored each of the thyroid nodules and designed a scoring system to classify thyroid nodules in terms of their class probabilities calculated by RF. Our score system could (i) standardize categorical reporting system and make ultrasonic report objective; (ii) quantize the description of the US finding indexes and provide helpful clinician guidelines in classifying the nodules, stratifying the risk of thyroid tumors, selecting patients to surgery or providing appropriate follow-up; (iii) significantly reduce the misdiagnosis after summarizing a large number of clinicians’ experience.

The malignancy-risk score computed by the RF algorithm conferred higher risk to malignant nodules as well as a lower risk to benign nodules rather than the number of suspicious characteristics; and then classified nodules into several diagnostic categories, each of which was associated with different cancer risks, ranging from benign to high suspicion. Therefore, clinicians or patients could get a definite possibility for malignancy of thyroid tumors through our presented scoring system.

“Hypoechoicinity” is a qualitative term and cannot give a piece of absolute objective information on the degree of echogenicity [27]. Considering the difference between the imaging of the diagnostic scanner and the subjective diagnosis of the radiologists, we used the relatively scientific echo ratio to unify the traditional echo intensity and quantify it. Patients with thyroid nodules often had diffuse thyroid lesions, and the level of glandular echo greatly changed. Accordingly, we divided the light and dark values in the image by the number of the anterior cervical anterior muscles with the echo level as the echo ratio parameter. At the same time, the quantified nodule echo values allowed us to further search for diagnostic cutpoint to substitute for the traditional diagnostics with hypoecho, equal echo, and hyperecho. Hypoechogenicity was operatively defined as the echogenicity ratio of less than or equal to 1.3 in our study, which was open to debate.

In our study, all nodules had been surgically diagnosed to be benign and malignant, helping us to evaluate the performance of classifiers. But this also led to sampling bias since nodules with a relatively higher risk of malignancy were usually recommended for surgery regardless of true benign, which directly led to benign nodules with multiple malignant characteristics in our samples. For example, there is no cystic nodule, which is one of the benign characteristics of thyroid nodules [10]. Therefore, it is difficult for radiologists or the computerized systems to correctly diagnose such benign nodules. As a result, the rate of misdiagnosis is usually high. However, the RF still performs better than other methods regardless of the disease and abnormal nodules. The diagnosis of abnormal nodules needs to be very careful since they may also be cancerous. Consequently, it can be categorized as borderline and recommended to FNA biopsy. In addition, Table 3 shows that the high prevalence of malignancy may affect the accuracy of the prediction for benign nodules, thus leading to the low NPV of classifiers for RF: 61.2%, LR: 60.0%, SVM: 58.2%, NET: 60.8%, ELM: 52.4%, KNN: 51.7%, NB: 56.9%, ADAB: 62.9%, LOG: 56.5% and LDA: 55.6%, respectively.

The limitation of this paper includes without considering real-time elastography data [12], interactions among the considered US characteristics, and outlier detections.

## Conclusions

We detected the US indicative characteristics of malignancy in thyroid nodules and designed a practical classifier scheme based on these characteristics to quantize the risk of malignancy. It could standardize the categorical reporting system and objectively make an ultrasonic report as well as simplify the description of the US characteristics by radiologists. The scoring system can be used to predict the risk of malignancy and guide the management decisions so as to reduce the number of unnecessary biopsy for benign nodules. In view of the fact that the proposed LLR together with RF performs better than other methods in identifying malignancy, especially for abnormal nodules, in terms of risk scores, we recommend the usage of the LLR together with RF method in applications.

## Data Availability

All data generated or analyzed during this study are included in this published article. Please contact the author for the code of the software and the documentation.

## Abbreviations

TI-RADS: Thyroid Imaging Reporting and Data System
RF: Random Forest
ER: the Echogenicity Ratio
TP: True positives
TN: True negatives
FP: False positives
FN: False negatives
LLR: Logistic Lasso Regression
ROC: Receiver operating characteristic curve
SEN: Sensitivity
SPE: Specificity
PPV: Positive predictive value
NPV: Negative predictive value
AUC: Area under curve
SVM: support vector machine
NET: neural network
KNN: K-nearst neighborhood
NB: Naive Bayesian
ADAB: Adaptive boosting
LOG: L^2^-logistic regression
LDA: Linear discriminant analysis
ELM: Extreme learning machine
FNA biopsy: Fine Needle Aspiration biopsy.
